# Involvement of amylin B-H_2_S-connexin 43 signaling pathway in vascular dysfunction and enhanced ischemia–reperfusion-induced myocardial injury in diabetic rats

**DOI:** 10.1042/BSR20194154

**Published:** 2020-06-08

**Authors:** Xiaoyong Liu, Rui Yang, Wenwei Bai, Xiang Xu, Feng Bi, Yingzheng Hao, Qishi Yang, Hu Li

**Affiliations:** 1 Department of Cardiovascular, The Second Affiliated Hospital of Kunming Medical University, Kunming, Yunnan, 650101, China; 2 Forensic medicine institution of Kunming Medical University, Kunming, Yunnan, 650500, China

**Keywords:** diabetes mellitus, ischemia, H2S, amylin, connexin 43

## Abstract

The present study was designed to investigate the role of amylin, H_2_S, and connexin 43 in vascular dysfunction and enhanced ischemia–reperfusion (I/R)-induced myocardial injury in diabetic rats. A single dose of streptozotocin (65 mg/kg) was employed to induce diabetes mellitus. After 8 weeks, there was a significant decrease in the plasma levels of amylin, an increase in I/R injury to isolated hearts (increase in CK-MB and cardiac troponin release) on the Langendorff apparatus. Moreover, there was a significant impairment in vascular endothelium function as assessed by quantifying acetylcholine-induced relaxation in norepinephrine-precontracted mesenteric arteries. There was also a marked decrease in the expression of H_2_S and connexin 43 in the hearts following I/R injury in diabetic rats. Treatment with amylin agonist, pramlintide (100 and 200 µg/kg), and H_2_S donor, NaHS (10 and 20 μmol/kg) for 2 weeks improved the vascular endothelium function, abolished enhanced myocardial injury and restored the levels of H_2_S along with connexin 43 in diabetic animals. However, pramlintide and NaHS failed to produce these effects the presence of gap junction blocker, carbenoxolone (20 and 40 mg/kg). Carbenoxolone also abolished the myocardial levels of connexin 43 without affecting the plasma levels of amylin and myocardial levels of H_2_S. The decrease in the amylin levels with a consequent reduction in H_2_S and connexin 43 may contribute to inducing vascular dysfunction and enhancing I/R-induced myocardial injury in diabetic rats.

## Introduction

Diabetes mellitus is a metabolic disorder, which is characterized primarily by a disturbance in glucose metabolism. Long-standing diabetes is associated with a large number of complications, including interference in the cardiovascular system [1]. There have been studies showing that long-standing diabetes enhances the extent of myocardial injury in response to ischemia–reperfusion [2]. The mortality rate of myocardial infarction patients suffering from diabetes mellitus is about 2–4 times higher in comparison with non-diabetic patients [3,4]. Therefore, there a need for identifying the mechanisms responsible for the enhancement in the myocardial injury during the diabetic state, and this may eventually help in the effective management of myocardial infarction in diabetic patients.

Amylin is a 37 amino acid peptide hormone, which is secreted along with insulin from the pancreas to regulate the glucose levels [5,6]. Hyperinsulinemia, usually associated with type II diabetes, is also associated with an increase in plasma amylin levels (hyperamylinemia) in diabetic patients [7,8]. The excessive circulating amylin may be deposited in different tissues and organs to produce cardiac dysfunction, cognitive impairment and renal disorders, etc. [9,10]. Since insulin and amylin are co-secreted and in most of cases of diabetes mellitus (particularly Type I diabetes mellitus), there is a decrease in insulin levels, therefore, hyperamylinemia may not be present in all diabetic patients. Indeed, there are studies documenting a decrease in the amylin levels in diabetes mellitus [11–13]. The importance of a decrease in amylin levels is emphasized by the studies showing that amylin analogs are useful in controlling glucose levels in diabetic mellitus [13]. Pramlintide is a synthetic amylin analog and is clinically employed to treat Type 1 and Type 2 diabetes [6,14]. Apart from regulating the glucose levels, amylin has also been proposed to reduce the β-amyloid burden and improve cognitive functions [15]. A recent study has shown that pramlintide improves learning and memory in an animal model of dementia [16]. It may be possible that the excessive circulating amylin (associated with hyperinsulinemia) may produce deleterious effects by depositing on the tissues/organs. However in diabetes-associated hypoamylinemia, exogenous amylin analogs may be useful in controlling glucose levels and associated diabetic complications. The dual role of amylin, i.e. protective or deleterious in diabetes, and Alzheimer’s disease have been discussed in a number of studies [15,17,18].

H_2_S is a gaseous neurotransmitter, and studies have shown that it regulates several biological functions in the body, including cardiovascular functions [19]. A large number of studies have demonstrated the effectiveness of H_2_S in alleviating ischemia–reperfusion-induced myocardial injury [20–22]. Moreover, a significant decrease in the levels of H_2_S has been reported in Type 2 diabetes patients and streptozotocin-treated diabetic rats [23], suggesting the possible relationship between a reduction of the levels of H_2_S and an increase in the extent of myocardial injury. Since both amylin [24,25] and H_2_S [26,27] are involved in regulating the insulin release; therefore, there is a possibility that there may be an interaction between amylin and H_2_S in diabetes mellitus. However, there is no study describing the interrelationship between amylin and H_2_S in diabetes mellitus and associated complications.

Connexin 43 is a gap junction alpha-1 protein, and it is a critical component of the gap junctions. Studies have documented that diabetes perturbs the intercellular communication and passage of electrical impulses across cells due to alteration in the gap junctions [28,29]. Furthermore, the disturbances in the gap junctions may contribute to the development of cardiovascular abnormalities, including myocardial infarction [30,31]. Thus, it may be hypothesized that diabetes-mediated changes in the myocardial expression of connexin 43 contribute to enhancing the extent of ischemia–reperfusion-induced myocardial injury. There have been studies showing that H_2_S attenuates cardiac dysfunction by restoring the myocardial expression of connexin 43 [32,33], suggesting the possible interrelationship between H_2_S and connexin 43 in diabetes-induced cardiovascular complications. Therefore, the present study was designed to investigate the role and interrelationship between amylin, H_2_S, and connexin 43 in inducing vascular dysfunction and enhancing ischemia–reperfusion-induced myocardial injury in diabetic rats.

## Material and methods

### Animals, drugs and chemicals

For the present study, male Wistar rats were employed, and the Institutional Animal Care and Committee of The Second Affiliated Hospital of Kunming Medical University approved the experimental animal studies, approval number: FEY-BG-15-1.1. The experiments were performed in the department of cardiovascular, The Second Affiliated Hospital of Kunming Medical University, Kunming, Yunnan, as per the ethical guidelines prescribed by the committee. The kits for estimating the levels of CK-MB and cardiac troponin T (cTnT) were procured from Jiancheng Reagent Co, Nanjing, China. The ELISA kits employed in the present study included connexin 43 (LifeSpan BioSciences, U.S.A.) and amylin (LifeSpan BioSciences, U.S.A.). The doses of water-soluble, non-peptide amylin agonist, pramlintide [16], H_2_S donor, NaHS [34], and gap-junctional blocker, carbenoxolone (20 mg and 40 mg/kg) [35] were selected on the basis of previous studies.

### Ischemia–reperfusion injury using the langendorff system

The rats were killed by cervical dislocation, and their hearts were rapidly excised to mount on the Langendorff apparatus. The hearts were perfused retrogradely with Kreb’s Henseleit solution (physiological solution) at 37°C [36,37]. The inflow of physiological solution to the heart was stopped to produce global ischemia for 30 min, and after that, reperfusion was instituted by restoring the flow of the physiological solution to the heart for 120 min. The coronary perfusate (the physiological solution after passing through the coronary arteries) was collected to estimate biomarkers of myocardial injury.

### Experimental protocol

The following experimental groups were included in the present study to meet the objectives of the study, and each experimental group comprises eight animals:

**Group I, Non-diabetic, non-ischemic control:** Non-diabetic rats were not subjected to ischemia–reperfusion injury. The hearts were isolated for biochemical analysis.
**Group II, Normal rats:** Normal non-diabetic animals were employed, and the hearts from these rats were subjected to 30 min of global ischemia and 120 min of reperfusion on the Langendorff apparatus as described above.
**Group III, Diabetic rats:** A single dose of streptozotocin (65 mg/kg i.p.) was injected to induce diabetes mellitus. These rats were kept for 8 weeks, and after the end of the stipulated time, the hearts were isolated and subjected to 30 min of global ischemia and 120 min of reperfusion on the Langendorff apparatus, as described above. The mesenteric arteries were isolated, and vascular function was assessed.
**Groups IV and V, Pramlintide (100 and 200 µg/kg)-treated diabetic rats:** Pramlintide (100 µg/kg) and pramlintide (200 µg/kg) were administered in diabetic rats in the respective IV and V groups, for the last 2 weeks. The rest of the procedure was the same as group III.
**Groups VI and VII, NaHS (10 and 20 μmol/kg)-treated diabetic rats:** NaHS (10 μmol/kg *i.p.*) and NaHS (20 μmol/kg *i.p.*) were administered in diabetic rats in the respective groups VI and VII for the last 2 weeks. The rest of the procedure was the same as group III.
**Groups VIII and IX, Carbenoxolone (20 and 40 mg/kg) and pramlintide (200 µg/kg)-treated diabetic rats:** Carbenoxolone (20 mg/kg *i.p.*) or carbenoxolone (40 mg/kg *i.p.*) was co-administered with pramlintide (200 µg/kg) in diabetic rats in the respective, VIII and IX groups for the last 2 weeks. The rest of the procedure was the same as group III.
**Groups X and XI, Carbenoxolone (20 and 40 mg/kg) and NaHS (20 μmol/kg)-treated diabetic rats:** Carbenoxolone (20 mg/kg *i.p.*) or carbenoxolone (40 mg/kg *i.p.*) was co-administered with NaHS (20 μmol/kg) in diabetic rats in the respective, X and XI groups for the last 2 weeks. The rest of the procedure was the same as group III.

### Assessment of ischemia–reperfusion-induced myocardial injury

The extent of myocardial injury was assessed by measuring the well-known biomarkers of myocardial injury, i.e., creatine kinase (CK-MB) and cardiac troponins (cTnT). The levels of these biomarkers were determined in the coronary perfusate immediately before subjecting to global ischemia (basal) and after instituting reperfusion. These levels of biochemical parameters were assessed by employing commercially available diagnostic kits. The extent of myocardial injury was assessed.

### Vascular endothelial function

After 8 weeks of a single dose of streptozotocin injection, the mesenteric arteries were removed from rats, and vascular endothelial functionality was determined. In short, the small ring segments (approximately 2 mm length) of mesenteric arteries were cut and mounted on the Myograph System. The endothelium-dependent vascular relaxation function was assessed in norepinephrine (10^−5^ M)-precontracted ring segments. These pre-contracted ring segments were allowed to relax by the cumulative addition of acetylcholine, and pIC50 was calculated, which served as an indirect measure of endothelium-dependent relaxation. pIC50 was defined as a negative log molar concentration (−log*M*) of acetylcholine that produced 50% relaxation in fully contracted arterial rings [38].

### Blood glucose levels

The blood glucose levels were measured in rats of different experimental groups before instituting ischemia–reperfusion injury. The rats were killed, and the blood was collected to measure different biochemical parameters, including the blood glucose levels.

### Quantification of H_2_S, amylin and connexin levels

The levels of amylin were measured in the blood before subjecting to ischemia–reperfusion injury. The blood was removed at the time of heart isolation. The levels of H_2_S in the heart homogenate were quantified colorimetrically using sodium hydrosulfide hydrate as standard [39–41]. The quantification of amylin in the blood and phosphorylated forms of connexin 43 in the homogenized mixture of heart was done using commercially available ELISA kits.

### Statistical analysis

The data of the present study were presented in the form of mean ± standard deviation (S.D.). The results of CK-MB and cTnT were analyzed using two way repeated measure ANOVA, considering the time (as samples were taken at two different time intervals) as one factor and treatment with different drugs as the second factor. All other results were analyzed using one-way ANOVA considering treatment with different drugs as the factor. After that, the multiple comparisons between the different groups were made using Tukey’s *post hoc* test. The statistical significance was calculated by fixing *P*<0.05.

## Results

### Diabetes exacerbates ischemia–reperfusion-induced myocardial injury

**Figure 1:**
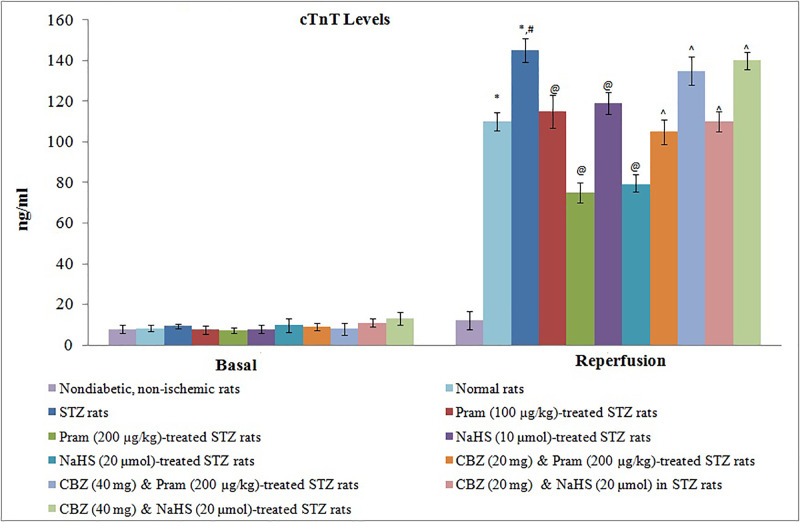
Effect of Different Interventions on the release of cTnTEffect of Different Interventions on the release of cTnT The modulatory effect of streptozotocin, pramlintide, NaHS, and carbenoxolone on ischemia–reperfusion-induced increase in cTnT release from the isolated hearts into coronary effluent, which were measured immediately before subjecting to ischemia (basal) and immediately after starting reperfusion**.** The values were presented in mean ± S.D. Two way repeated measure ANOVA was applied for comparing different groups and for time factor, F (1, 154) = 1345.2; for treatment factor, F (10, 154) = 457.6. *P<0.05 vs. basal; #*P*<0.05 vs. normal rats during reperfusion; @*P*<0.05 vs*.* STZ rats during reperfusion; ∧*P*<0.05 vs. Pram (high dose)/NaHS (high dose)-treated STZ rats; CBZ, Carbenoxolone; Pram, Pramlintide; STZ, Streptozotocin.

**Figure 2:**
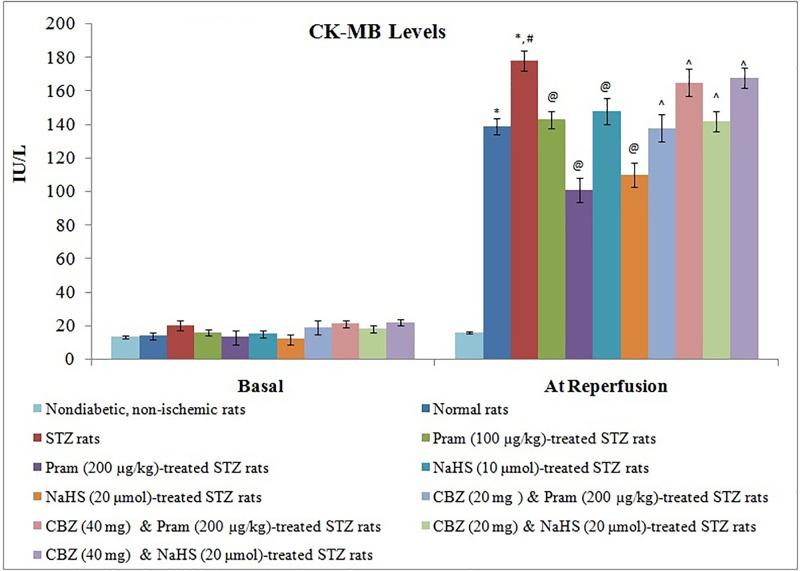
Effect of Different Interventions on the release of CK-MB The modulatory effect of streptozotocin, pramlintide, NaHS, and carbenoxolone on ischemia–reperfusion-induced increase in CK-MB release from the isolated hearts into coronary effluent, which were measured immediately before subjecting to ischemia (basal) and immediately after starting reperfusion. The values were presented in mean ± S.D. Two way repeated measure ANOVA was applied for comparing different groups, and for the time factor, F (1, 154) = 1465.8; for treatment factor, F (10, 154) = 558.1. *P<0.05 vs. basal; #*P*<0.05 vs. normal rats during reperfusion; @*P*<0.05 vs. STZ rats during reperfusion; ∧*P*<0.05 vs*.* Pram (high dose)/NaHS (high dose)-treated STZ rats; CBZ, Carbenoxolone; Pram, Pramlintide; STZ, Streptozotocin.

In non-diabetic rats, 30 min of global ischemia and 120 min of reperfusion led to myocardial injury as assessed by a significant increase in the levels of cTnT (Figure 1) and CK-MB (Figure 2) in the coronary perfusate collected during the reperfusion phase in comparison with corresponding basal levels (before ischemia). The extent of CK-MB and cTnT release was more significant from the hearts of diabetic rats in comparison with non-diabetic rats suggesting that the extent of myocardial injury was increased during the state of diabetes mellitus.

### Influence of different pharmacological interventions on diabetes-induced exacerbation of ischemia–reperfusion-induced myocardial injury

Treatment of diabetic rats with pramlintide (100 and 200 µg/kg) and NaHS (10 μmol/kg and 20 μmol/kg) for 2 weeks significantly abrogated diabetes-induced exacerbation of myocardial injury in ischemia–reperfusion-subjected hearts. There was a significant reduction in the release of CK-MB (Figure 1) and cTnT (Figure 2) in the coronary perfusate in pramlintide and NaHS-treated diabetic rats. However, co-administration of carbenoxolone with pramlintide or NaHS significantly abolished the cardioprotective effects of these pharmacological agents. In other words, pramlintide or NaHS failed to exert their cardioprotective effects on ischemia–reperfusion-subjected hearts of diabetic rats in the presence of carbenoxolone.

### Influence of different pharmacological interventions on blood glucose levels

**Figure 3:**
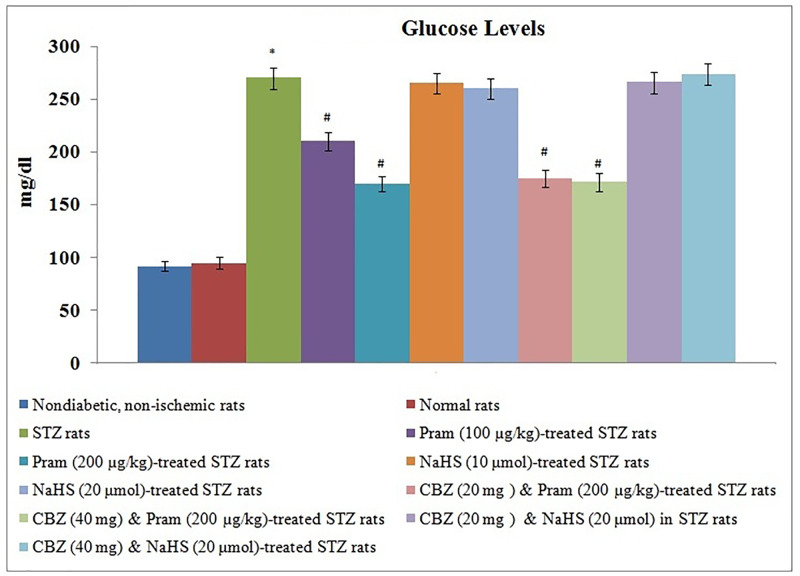
Effect of Different Interventions on the blood glucose levels The modulatory effect of streptozotocin, pramlintide, NaHS, and carbenoxolone on the blood glucose levels, measured before instituting ischemia-reperfusion injury**.** The values were presented in mean ± S.D. One way ANOVA was applied for comparing different groups and F (10,77) = 689.2. *P<0.05 vs. normal rats; #*P*<0.05 vs. STZ rats; CBZ, Carbenoxolone; Pram, Pramlintide; STZ, Streptozotocin.

There was a significant rise in the fasting blood glucose levels in diabetic rats in comparison with non-diabetic rats suggesting the state of diabetes mellitus in streptozotocin-administered rats. Treatment with pramlintide significantly lowered blood glucose levels. However, other pharmacological modulators including NaHS and carbenoxolone did not modulate the blood glucose levels in diabetic rats in a significant manner (Figure 3).

### Influence of different pharmacological interventions on H_2_S, amylin, and p-connexin 43 levels

**Figure 4:**
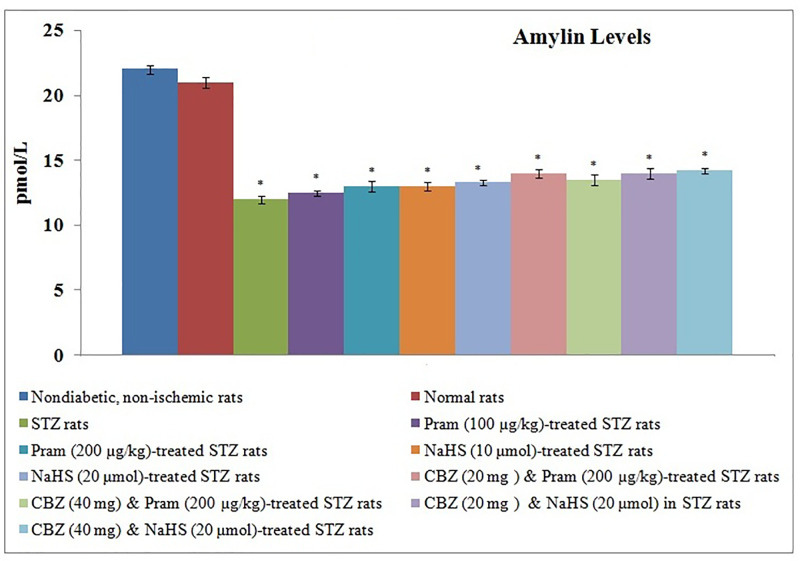
Effect of Different Interventions on the amylin levels The modulatory effect of streptozotocin, pramlintide, NaHS, and carbenoxolone on the plasma amylin levels, measured before instituting ischemia-reperfusion injury**.** The values were presented in mean ± S.D. One way ANOVA was applied for comparing different groups and F (10,77) = 241.2. *P<0.05 vs. normal rats; CBZ, Carbenoxolone; Pram, Pramlintide; STZ, Streptozotocin;*P<0.05 vs. normal.

**Figure 5:**
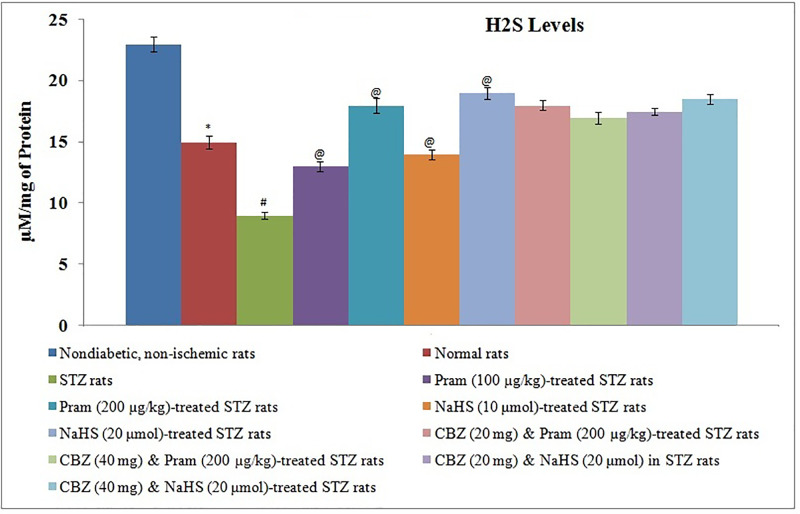
Effect of Different Interventions on the release of H2S levels The modulatory effect of streptozotocin, pramlintide, NaHS, and carbenoxolone on the H_2_S concentration in the myocardium following 30 min of ischemia and 120 min of reperfusion**.** The values were presented in mean ± S.D. One way ANOVA was applied for comparing different groups and F (10,77) = 351.5.*P<0.05 vs. non-diabetic non-ischemic rats; #*P*<0.05 vs. normal rats; @ vs. STZ rats; CBZ, Carbenoxolone; Pram, Pramlintide; STZ, Streptozotocin.

**Figure 6:**
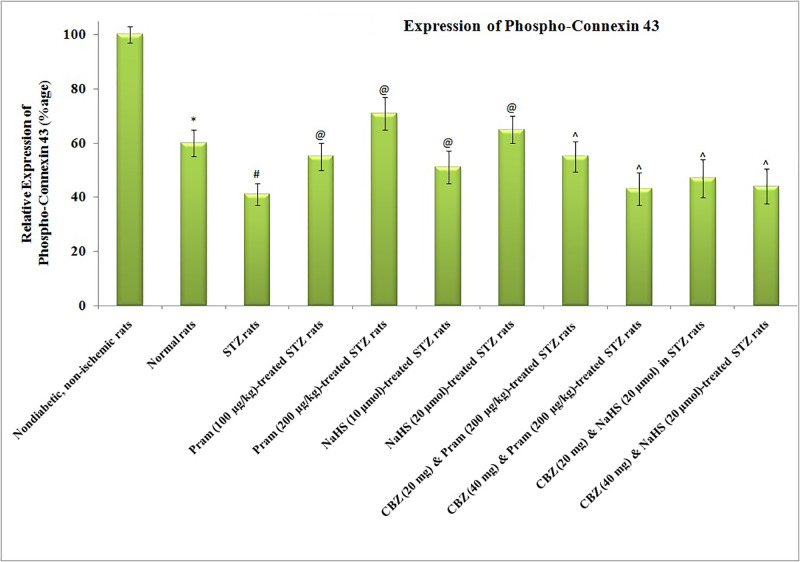
Effect of Different Interventions on the release of phospho-connexin 43 levels The modulatory effect of streptozotocin, pramlintide, NaHS, and carbenoxolone on the expression of connexin 43 in the hearts following 30 min of ischemia and 120 min of reperfusion. The myocardial levels of connexin 43 were normalized to β-actin and thereafter, the data were normalized to non-diabetic, non-ischemic control. The values were presented in mean ± S.D. One way ANOVA was applied for comparing different groups and F (10,77) = 393.4. *P<0.05 vs. non-diabetic, non-ischemic, basal; #*P*<0.05 vs. normal; @*P*<0.05 vs. diabetes; ∧*P*<0.05 vs. Pram (high dose)/NaHS (high dose)-treated STZ rats; CBZ, Carbenoxolone; Pram, Pramlintide; STZ, Streptozotocin.

There was a significant reduction in the plasma levels of amylin in diabetic rats, measured before instituting ischemia–reperfusion injury, in comparison with non-diabetic rats (Figure 4). Treatment with pramlintide and NaHS did not modulate the levels of amylin in diabetic rats. Administration of carbenoxolone did not alter the plasma levels of amylin in pramlintide and NaHS-treated diabetic rats. There was a significant reduction in the concentration of H_2_S (Figure 5) and the levels of phosphorylated forms of connexin 43 protein (Figure 6) in ischemia–reperfusion-subjected hearts, isolated from diabetic and non-diabetic rats. However, the reduction in H_2_S concentration and p-connexin 43 levels was more significant in diabetic rats in comparison with non-diabetic rats. Treatment with pramlintide and NaHS led to the restoration of H_2_S concentration and p-connexin 43 levels in diabetic rats. Co-administration of carbenoxolone abolished the restorative effects of pramlintide and NaHS on the p-connexin 43 levels, without any significant effect on the H_2_S concentration in diabetic rats.

### Influence of different pharmacological interventions on the vascular endothelium function

In non-diabetic rats, the cumulative addition of acetylcholine led to complete relaxation of norepinephrine-precontracted mesenteric arteries indicating the normal endothelium function in non-diabetic rats. On the other hand, the cumulative addition of acetylcholine produced partial relaxation in norepinephrine-precontracted mesenteric arteries isolated from diabetic rats indicating the existence of vascular endothelium dysfunction (Table 1). Considering pIC50 as a negative log molar concentration of acetylcholine that produces 50% relaxation in fully contracted arterial rings, the pIC50 values were used as an indirect measure of endothelium-dependent relaxation. The pIC50 value was significantly lower in diabetic rats as compared with non-diabetic rats indicating that a higher concentration of acetylcholine was required to produce comparable relaxation in the mesenteric arteries of diabetic rats (Table 2). Treatment with pramlintide (100 and 200 µg/kg) and NaHS (10  and 20 μmol/kg) for 2 weeks significantly increased pIC50 value in the diabetic group. It indicates that there was an improvement in vascular functionality, and the cumulative addition of acetylcholine was able to produce complete relaxation in norepinephrine-precontracted mesenteric arteries. However, co-administration of carbenoxolone with pramlintide or NaHS significantly abolished the vascular endothelium improving the functions of these pharmacological agents.

**Table 1:** Percentage of contraction remaining in the mesenteric arteries after cumulative addition of acetylcholine (−log*M*) in norepinephrine (NE)-precontracted arteries, isolated from non-diabetic and diabetic animals (*n*=8 for each group)

Serial No.	Log*M* Concentration of Ach	Percentage of contraction remaining in arteries after cumulative addition of acetylcholine in NE-precontracted arteries									
		Normal	STZ rats	Pram (100 µg/kg) -treated STZ rats	Pram (200 µg/kg) -treated STZ rats	NaHS (10 μmol/kg) -treated STZ rats	NaHS (20 μmol/kg) -treated STZ rats	CBZ (20 mg/kg) & pram (200 µg/kg) -treated STZ rats	CBZ (40 mg/kg) & pram (200 µg/kg) -treated STZ rats	CBZ (20 mg/kg) & NaHS (20 μmol/kg) -treated STZ rats	CBZ (40 mg/kg) & NaHS (20 μmol/kg) -treated STZ rats
1	−9.0	94.2 ± 3.9	98.9 ± 3.8	98.1 ± 3.1	96.5 ± 2.7	98.8 ± 3.0	95.8 ± 2.4	97.6 ± 2.5	98.4 ± 2.1	97.8 ± 2.9	98.3 ± 2.1
2	−8.5	82.1 ± 3.3	91.6 ± 3.2	89.1 ± 2.7	87.2 ± 2.9	88.4 ± 2.4	86.1 ± 2.3	89.2 ±2.6	90.3 ± 1.9	88.1 ± 1.8	89.9 ± 2.0
3	−8.0	73.2 ± 3.2	85.6 ± 3.1	81.2 ± 2.9	77.4 ± 2.8	80.4 ± 2.1	76.3 ± 2.4	80.4 ± 2.3	84.2 ± 1.6	79.3 ± 2.1	83.3 ± 2.5
4	−7.5	59.2 ± 2.4	77.4 ± 2.9	70.3 ± 2.5	64.1 ± 2.0	69.2 ± 2.6	63.6 ± 2.9	69.3 ±2.2	74.0 ± 2.8	68.9 ± 2.1	73.6 ± 2.5
5	−7.0	44.1 ± 2.7	71.3 ± 2.6	60.3 ± 2.8	53.1 ± 1.9	58.1 ± 2.8	52.1 ± 1.9	63.1 ± 2.0	68.4 ± 2.6	62.1 ± 1.8	67.2 ± 1.6
6	−6.5	32.6 ± 2.7	62.3 ± 2.1	53.2 ± 2.1	42.0 ± 2.5	52.2 ± 2.7	41.2 ± 1.5	52.3 ± 2.4	64.1 ±2.6	51.3 ± 2.1	63.5 ±2.0
7	−6.0	17.3 ± 2.7	57.1 ± 2.1	37.1 ± 2.8	24.7 ± 1.8	35.1 ± 1.7	23.5 ± 1.2	39.5 ± 1.7	53.1 ± 1.9	38.1 ± 1.9	51.1 ± 1.5
8	−5.5	8.3 ± 0.9	51.3 ± 2.3	21.2 ± 1.8	14.2 ± 1.2	20.5 ± 1.1	12.9 ± 1.0	28.8 ±2.0	46.4 ± 2.4	27.6 ±2.3	44.4 ± 2.1
9	−5.0	1.9 ± 0.6	45.2 ± 2.7	13.1 ± 1.1	6.3 ± 0.7	11.5 ± 0.8	5.8 ±0.9	21.1 ± 1.9	35.2 ± 2.1	20.5 ± 1.3	33.2 ± 2.0

The values were expressed as mean ± S.D. Ach, Acetylcholine; CBZ, Carbenoxolone; Pram, Pramlintide; STZ: streptozotocin. The values from nondiabetic, nonischemic rats were not denoted as the values were very similar to normal rats.

**Table 2:** Effect of different pharmacological interventions on pIC50 (−log*M*) values of acetylcholine in norepinephrine-precontracted mesenteric arteries in different experimental groups with *n*=8 in each group

Serial No	Experimental Groups	pIC_50_ (−log*M*)
1.	Nondiabetic, non-ischemic rats	7.20± 1.34
2.	Normal rats	7.15 ± 1.42
3.	STZ rats	5.43 ± 1.82*
4.	Pramlintide (100 µg/kg)-treated STZ rats	6.37 ± 1.51^†^
5.	Pramlintide (200 µg/kg)-treated STZ rats	6.81 ± 1.62^†^
6.	NaHS (10 μmol/kg)-treated STZ rats	6.41 ± 1.45^†^
7.	NaHS (20 μmol/kg)-treated STZ rats	6.89 ± 1.85^†^
8.	Carbenoxolone (20 mg/kg) and pramlintide (200 µg/kg)-treated STZ rats	6.32 ± 1.50
9.	Carbenoxolone (40 mg/kg) and pramlintide (200 µg/kg)-treated STZ rats	5.70 ± 2.20^‡^
10.	Carbenoxolone (20 mg/kg) and NaHS (20 μmol/kg)-treated STZ rats	6.34 ± 1.50
11.	Carbenoxolone (40 mg/kg) and NaHS (20 μmol/kg)-treated STZ rats	5.72 ± 1.90^‡^

The values were expressed as mean ± S.D. One way ANOVA was applied for comparing different groups and F (10,77) = 125.5. *P<0.05 vs. normal rats; †*P*<0.05 vs. STZ rats; ‡*P*<0.05 vs. pramlintide (high dose)/NaHS (high dose)-treated STZ rats.

## Discussion

In the present study, 30 min of ischemia and 120 min of reperfusion led to a significant heart injury in normal non-diabetic rats as assessed by an increase in the release of including CK-MB and cTnT in the coronary perfusate. These are well-documented biomarkers of myocardial damage, and these are released from the heart to the coronary perfusate due to the rupturing of the plasma membrane [37,42]. Institution of global ischemia and reperfusion in isolated rat hearts also led to the significant myocardial injury in diabetic rats. Comparatively, the extent of myocardial injury was statistically more severe in diabetic rats than in the same age group of non-diabetic rats. Moreover, there was a vascular endothelium dysfunction in diabetic rats assessed in terms of a decrease in relaxation on the cumulative addition of acetylcholine in norepinephrine-precontracted mesenteric arterial rings. The presence of diabetes mellitus was confirmed in streptozotocin-injected rats by determining the increase in the blood glucose levels before instituting global ischemia–reperfusion injury to rat hearts. The blood glucose levels were significantly higher in streptozotocin-injected rats in comparison with the corresponding same age group of non-diabetic rats. The increase in the extent of ischemia–reperfusion induced myocardial injury during hyperglycemic conditions in obese Zucker diabetic rats [43] and streptozotocin-treated rats is well documented [44,45]. Moreover, the presence of vascular endothelium dysfunction in streptozotocin-treated rats is also documented [46].

In the present study, there was a decrease in the blood levels of amylin in diabetic rats, measured before instituting global ischemia–reperfusion injury in comparison with non-diabetic rats. There have been studies showing a decrease in the levels of amylin during diabetes mellitus [11,12]. The significant reduction in the amylin levels probably suggests that it may be one of the factors contributing to increased myocardial injury and vascular dysfunction during the diabetic state. To further explore the role of amylin, diabetic rats were treated with amylin analog (pramlintide) for two weeks before subjecting to ischemia–reperfusion injury. Treatment of diabetic rats with pramlintide produced cardioprotective effects, and the extent of myocardial injury was significantly abolished in these rats. Moreover, there was a marked improvement in the vascular endothelium function in pramlintide-treated diabetic rats. These findings further support the hypothesis that the decrease in the levels of amylin and consequent decreased activation of amylin receptors may be responsible for enhanced myocardial injury and vascular dysfunction in diabetic rats. Accordingly, exogenous administration of amylin analog or pharmacological activator of amylin receptors may be potentially employed as therapeutic agents in alleviating the increased tendency of ischemia–reperfusion-induced myocardial injury in diabetic patients. There have been studies showing the beneficial effects of pramlintide in improving learning and memory in an animal model of Alzheimer’s disease [16]. To best of our knowledge, it is the first study showing the potential of pramlintide in preventing vascular dysfunction and exaggerated form of ischemia–reperfusion-induced myocardial injury in diabetic rats.

Along with the decrease in the levels of amylin, there was also a significant decline in the H_2_S concentration and phospho-connexin 43 levels in the hearts of diabetic rats, measured after ischemia–reperfusion injury. There have been studies showing that the concentration of H_2_S is decreased during the state of diabetes [23], and a decrease in H_2_S concentration contributes to inducing myocardial injury [47]. Moreover, studies have also shown the decrease in connexin 43 levels during diabetic conditions [48] and restoration of connexin 43 has been reported to preserve myocardial functions and prevent injury [49,50]. Along with the decrease in myocardial injury, treatment with amylin analog restored the concentration of H_2_S and connexin 43 levels in the myocardium of diabetic rats. Accordingly, it may be proposed that the beneficial effects of amylin analog in attenuating ischemia–reperfusion injury in the diabetic state may be secondary to an increase H_2_S concentration and connexin 43 expression. It is the first study describing that amylin analog may increase the H_2_S concentration to produce cardio- and vasculo-protective effects in diabetic rats.

Considering that the possible role of the decrease in H_2_S concentration in the diabetic state in enhancing myocardial injury and vascular dysfunction, diabetic rats were treated with H_2_S donor (NaHS) and, subsequently, the effects of ischemia–reperfusion injury were assessed on isolated rat hearts. The results depicted that there was a significantly lesser degree of myocardial injury and vascular dysfunction in NaHS-treated diabetic rats, and the results were comparable to amylin analog-treated animals. There have been studies showing that hydrogen sulfide preconditions and protects diabetic mouse heart against ischemia–reperfusion injury [51,52]. Like amylin analog, treatment with NaHS also restored the expression of connexin 43 in the myocardium of diabetic rats suggesting that connexin 43 may be the common target of amylin analog and H_2_S-mediated protective effects. It has been reported that treatment with H_2_S improves the expression of connexin 43 in the heart [32,40]. However, treatment with NaHS did not modulate the plasma levels of amylin in diabetic rats suggesting that H_2_S may be the downstream target of amylin. Therefore, it may be proposed that amylin analog may increase the H_2_S concentration in the myocardium, which in turn may increase the expression of myocardial connexin 43 to attenuate ischemia–reperfusion injury in diabetic rats.

To further elucidate the inter-relationship among amylin, H_2_S and connexin 43, a gap junction blocker, i.e. carbenoxolone, was co-administered with amylin analog and H_2_S donor in diabetic rats. Interestingly, amylin analog, as well as H_2_S donor, failed to exert cardioprotection in diabetic rats in the presence of gap junction blocker supporting the above contention that amylin analog and H_2_S donor produce cardioprotection by acting through a common target, i.e. connexin 43. Moreover, carbenoxolone did not alter the plasma levels of amylin and the myocardial levels of H_2_S in either of amylin analog and H_2_S donor-treated diabetic rats. In the present study, the role of H_2_S and connexin 43 in myocardial injury was explored using pharmacological interventions and by their direct measurement in the heart. However, their role in diabetes-induced vascular dysfunction was delineated using pharmacological interventions. Future studies may be designed to strengthen the role of H_2_S and connexin 43 in vascular endothelial dysfunction by their direct measurement in that tissue.

## Conclusion

The decrease in the plasma levels of amylin with a consequent decrease in H_2_S levels and connexin 43 expression in the heart may contribute to enhancing ischemia–reperfusion-induced myocardial injury in diabetic rats. Amylin analog and H_2_S donors may be useful therapeutic agents in attenuating long-standing diabetes mellitus-induced vascular endothelium dysfunction and enhanced ischemia–reperfusion-induced myocardial injury. It is possible that amylin analog may increase the H_2_S levels, which in turn may increase the expression of connexin 43 to produce beneficial effects on the cardiovascular system in diabetic rats.

## Abbreviations

Ach: Acetylcholine
CBZ: Carbenoxolone
CK-MB: creatine kinase
cTnT: cardiac troponin T
I/R: ischemia–reperfusion
Pram: Pramlintide
STZ: streptozotoci
